# A General Model for Toxin-Antitoxin Module Dynamics Can Explain Persister Cell Formation in *E. coli*

**DOI:** 10.1371/journal.pcbi.1003190

**Published:** 2013-08-29

**Authors:** Lendert Gelens, Lydia Hill, Alexandra Vandervelde, Jan Danckaert, Remy Loris

**Affiliations:** 1 Applied Physics Research Group (APHY), Vrije Universiteit Brussel, Brussels, Belgium; 2 Molecular Recognition Unit, Department of Structural Biology, VIB, Brussels, Belgium; 3 Structural Biology Brussels, Department of Biotechnology, Vrije Universiteit Brussel, Brussels, Belgium; Stanford University, United States of America

## Abstract

Toxin-Antitoxin modules are small operons involved in stress response and persister cell formation that encode a “toxin” and its corresponding neutralizing “antitoxin”. Regulation of these modules involves a complex mechanism known as conditional cooperativity, which is supposed to prevent unwanted toxin activation. Here we develop mathematical models for their regulation, based on published molecular and structural data, and parameterized using experimental data for F-plasmid *ccdAB*, bacteriophage P1 *phd/doc* and *E. coli relBE*. We show that the level of free toxin in the cell is mainly controlled through toxin sequestration in toxin-antitoxin complexes of various stoichiometry rather than by gene regulation. If the toxin translation rate exceeds twice the antitoxin translation rate, toxins accumulate in all cells. Conditional cooperativity and increasing the number of binding sites on the operator serves to reduce the metabolic burden of the cell by reducing the total amounts of proteins produced. Combining conditional cooperativity and bridging of antitoxins by toxins when bound to their operator sites allows creation of persister cells through rare, extreme stochastic spikes in the free toxin level. The amplitude of these spikes determines the duration of the persister state. Finally, increases in the antitoxin degradation rate and decreases in the bacterial growth rate cause a rise in the amount of persisters during nutritional stress.

## Author Summary

Bacterial persistence plays an important role in many chronic infections. Persisters are subpopulations of bacteria which are tolerant to biological stresses such as antibiotics because they are in a dormant, non-dividing state. Toxin-antitoxin (TA) modules play a pivotal role in persister generation and bacterial stress response. These small genetic loci, ubiquitous in bacterial genomes and plasmids, code for a toxin that slows down or halts bacterial metabolism and a corresponding antitoxin that regulates this activity. In order to further unravel the intricate autoregulation of TA modules and their role in persister cell formation, we built stochastic models describing the transcriptional regulation including conditional cooperativity. This is a complex mechanism in which the molar ratio between both proteins determines whether the toxin will behave as a co-repressor or as a de-repressor for the antitoxin. We found that the necessary protein production and therefore the energetic cost decreases with increased binding site number. Finally, these models allow us to simulate the formation of persister cells through rare, stochastic increases in the free toxin level. We believe that our analysis provides a fresh view and contributes to our understanding of TA regulation and how it may be related to the emergence of persisters.

## Introduction

Stress response is an important aspect of the physiology of bacteria, allowing them to deal with a continuously changing environment and exposure to altering and fluctuating food sources as well as life-threatening chemicals such as antibiotics. Among the elements involved in bacterial stress response are the type II toxin-antitoxin (TA) modules [1], [2]. These are found in prokaryotes as pairs of genes encoding a protein that interferes with basic metabolism (the toxin) and its regulator (the antitoxin). The toxins display a variety of three-dimensional folds and biochemical activities: CcdB and ParE family members inhibit gyrase [3], [4], although via different molecular mechanisms. MazF toxins are structurally similar to CcdB but function as ribonucleases that degrade specific mRNAs and/or modify ribosomes [5]–[7]. RelE toxins, however, are structurally related to ParE but bind at the A site of the ribosome and degrade mRNAs in a translation-dependent manner [8], [9]. Other toxins such as HipA and Doc arrest translation without RNA degradation, for example through phosphorylation of elongation factor Tu [10]. A variety of biological roles have been attributed to TA modules ranging from molecular parasites over the stabilization of genetic elements (plasmids, introns and labile chromosomal segments) to altruistic suicide and the generation of persister cells.

Persisters are cells which exhibit multidrug tolerance, not because of a specific resistance mechanism like a mutation in an antibiotic target, but because they are in a dormant, slow-growing state. Cell-wall synthesis, translation and topoisomerase activity are slowed down in dormant cells, making it impossible for bactericidal antibiotics, whose targets are often implicated in these general metabolic processes, to kill the cells [11]. Persisters pre-exist in bacterial populations [12], they are subpopulations that allow survival of the bacterial colony in the case of severe environmental stresses. As such, they are involved in the multidrug tolerance of biofilms and the recalcitrance of bacterial infectious diseases [11]. As expression of TA toxins can bring cells in a “dormant state” or reversible stasis [13], TA modules have been linked to the development of persisters [14].

Each type of toxin is associated with one or more types of antitoxins, leading to a large number of TA families [15]. The antitoxins are typically two domain proteins consisting of a folded common DNA binding domain (helix-turn-helix, ribbon-helix-helix, AbrB fold, etc.) associated with an intrinsically disordered toxin-neutralizing segment that folds upon binding. Regulation of the toxin activity is achieved by balancing the synthesis and proteolytic degradation of the antitoxin [16]. Therefore TA modules are typically activated (for example during nutritional stress) by an increased activity of housekeeping proteases such as Lon and ClpXp [17], [18]. The neutralization function of the antitoxin is however not necessarily passive. In certain cases such as gyrase poisoning by CcdB, the intrinsically disordered domain of the antitoxin was shown to play an active role in reactivating the stalled gyrase molecules [19].

TA modules are further regulated at the transcription level by a mechanism termed “conditional cooperativity” [20]. Here the toxin acts as a co-repressor or anti-repressor depending on the ratio between toxin and antitoxin. When either an excess of toxin or antitoxin is available in the cell, transcription will occur until the cellular ratio is balanced and a repressing toxin-antitoxin complex is predominantly formed. Conditional cooperativity has been observed in all classic type II TA families where it was investigated, independent of the toxin or antitoxin fold, the operator size or the toxin target [21]–[24]. The molecular mechanisms leading to conditional cooperativity vary and involve a low-to-high affinity switch going from a repressing to a non-repressing toxin-antitoxin complex and/or steric exclusion principles [19], [23], [25].

Since the toxins interfere with the basic bacterial metabolism, free toxins can have an inhibitory effect on bacterial growth. This growth inhibition, in turn, can lead to changes in gene expression, as the RNA transcription rates and the protein dilution rates depend on the growth rate [26]. Although relevant results were obtained with models excluding these toxic effects (see for example [27]), it is clear that including the interaction of the toxin-antitoxin module with the host bacterium will lead to more realistic conclusions. The impact of gene circuits on host physiology can lead to drastic changes in the dynamics of the gene circuits themselves, as demonstrated by Tan *et al.* [28], who found that bistability in the expression of a mutant T7 RNA polymerase was caused by the reduction in growth rate due to the expression of this non-toxic protein. Furthermore, Nevozhay *et al.* recently showed how the interplay of individual cell growth rate and cellular memory jointly determine the overall cell population fitness in a bistable synthetic gene circuit when including variable division rates of single cells [29]. Several mathematical models have already been used in the study of persister cell formation. For example, Balaban *et al.* modeled the phenotypic switch between normally growing cell populations and persisters, discriminating two different types of persisters, one generated during the stationary phase and one which spontaneously arises during growth [12].

As TA modules are implicated in the formation of persister cells [14], [30], [31], the regulatory network of these systems has been modeled as the underlying cause of persister generation. Koh and Dunlop built a model for the *hipBA* TA module, including transcription, translation and repression of gene expression by the antitoxin and a toxin-antitoxin complex [27]. They argue that persistence is not caused by bistability, but by stochastic fluctuations in the expression of HipA and HipB, causing the free toxin level to exceed a threshold. The autoregulation of the *relBE* module was studied by Cataudella *et al.*, who found that conditional cooperativity prevents random toxin activation in growing cells and promotes fast translational recovery by quickly removing the free toxin after a period of starvation [32].

Although these publications significantly contributed to our understanding of persister cells and TA modules, we believe that a general modeling framework that includes conditional cooperativity and that is applicable to several toxin-antitoxin families could help to answer several of the remaining questions in this field, such as the role of multiple binding sites on the operator and the effect of toxin-dependent cell growth rate modulation. This paper presents a theoretical analysis of transcription regulation by conditional cooperativity based upon parameters available for the *ccdAB* (F-plasmid), *phd/doc* (bacteriophage P1) and *relBE* (*E. coli*) modules, three TA modules that are well characterized. We study both the molecular mechanism observed in the *relBE* TA module, where the binding sites on the operator are considered independent, and present the first mathematical model for the mechanism observed in the *ccdAB* and *phd/doc* TA modules, where an interaction between the different binding sites on the operator exists as chains of alternating toxins and antitoxins can be formed on the DNA.

## Results

### Two models to describe conditional cooperativity

**Figure 1:**
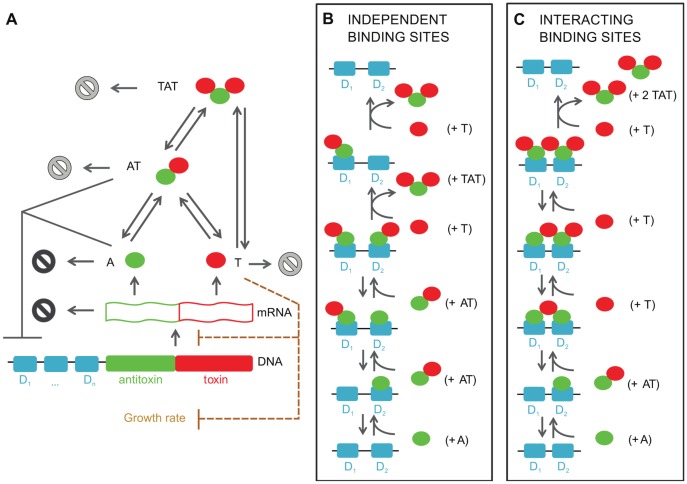
Toxin-antitoxin models for one or more binding sites on the operator based on the repression. (A) A toxin-antitoxin module typically consists of a promoter/operator region, followed by the genes for the antitoxin and the toxin. After transcription of the polycistronic mRNA, the toxin and antitoxin are translated. These proteins can form two non-toxic complexes, AT and TAT. The degradation (represented by 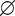) of antitoxin and mRNA is more intense (indicated in black) than degradation of toxin and both complexes AT and TAT, in which case the degradation rate corresponds to dilution by cell division. (B) Molecular mechanism of conditional cooperativity in the “independent binding sites” model as experimentally observed for *relBE*. Two AT complexes complexes can bind independently next to each other. Addition of a third toxin to form a TAT complex leads to release of this entity from the operator. (C) Molecular mechanism in the “interacting binding sites” model. In this case, as observed for *ccdAB* and *phd/doc*, the toxin has two binding sites for an antitoxin and is thus able to bridge two antitoxin dimers on the operator via a low and a high affinity interaction. Addition of an additional toxin leads to a switch from a low to a high affinity interaction, and the resulting TAT complex again is released from the operator.

We model TA modules based on all essential interactions in three well-studied systems: the F-plasmid *ccdAB*, bacteriophage P1 *phd/doc* and *E. coli relBE* operon (Figure 1). Common to these systems is that the toxin and antitoxin can form complexes with distinct stoichiometries and DNA binding properties. In the figure the free antitoxin (A) and the free toxin (T) correspond to the biologically relevant species and are typically dimers for the antitoxin, but can be monomers (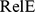, 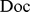) or dimers (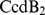) for the toxin, depending on the TA module considered. The AT complex (corresponding to the molecular species 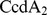-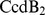, 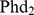-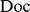 and 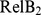-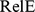) has a higher affinity for the operator sites than the isolated antitoxin. The TAT species consists of two toxins flanking a single antitoxin dimer - corresponding to 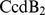-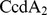-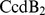, 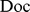-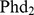-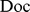 and 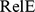-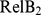-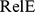 species. As the DNA binding properties of this species are dependent on the TA module considered, they will be discussed below. The TA operator has one or more binding sites (denoted 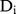 with 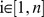 in Figure 1A) for A, AT and/or TAT. It is assumed that transcription is halted when at least one molecule (A) or complex (AT or TAT) is bound on the operator. When no proteins are bound on the operator, the genes coding for the toxin and antitoxin are transcribed. Translation of the mRNA leads to the creation of toxin and antitoxin. In TA modules the translation rate for the antitoxin has been found to be larger than the one for the toxin [17]. Therefore, when the toxin-antitoxin operator is being freely expressed, initially more antitoxin than toxin will be created. However, the antitoxin is degraded faster than the much more stable toxin, influencing the steady state toxin∶antitoxin ratio. The degradation of the antitoxins also extends to those within the complexes AT and TAT. Although the bound toxins protect the antitoxin from proteolytic degradation, this protection is not complete and the decay of antitoxin within complexes allows for the release of the attached toxins.

When it comes to the DNA binding interactions at the operator, there are fundamental differences between the three studied TA systems. The mechanism in the *relBE* system is the basis for the “independent binding site model” (Figure 1B). In this model, it is assumed that the binding sites on the operator behave independently, and either an individual antitoxin or a toxin-antitoxin complex can bind to each binding site. Conditional cooperativity is included in this model by ensuring that the AT complex has a higher affinity for the binding sites on the DNA than the antitoxin alone. Therefore, the toxin can act as a co-repressor for the antitoxin. Furthermore, we assume that the binding of an extra toxin to a DNA-bound AT complex will lead to the detachment of a TAT complex (shown in the final step of Figure 1B) from the promoter/operator, enabling mRNA transcription to proceed if all binding sites are unbound. The toxin can therefore function as a derepressor in the autoregulation of the operon by removing (through the formation of the secondary complex TAT) the bound proteins when the ratio of total toxin to total antitoxin (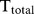
∶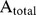) is high.

The second or “interacting binding site” model (Figure 1C) considers more complex binding processes on the DNA, experimentally observed for the *ccdAB* and the *phd/doc* modules [19], [23]. In this case, toxins can bridge different binding sites on the operator, forming a chain of alternating toxins and antitoxins. When the 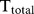
∶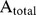 ratio increases, this complex can again be released from the DNA: An extra toxin comes in and the soluble TAT complexes are formed (shown in the final step of Figure 1C), as it is impossible for TAT complexes to occupy adjacent binding sites on the promoter/operator due to steric clashes.

Finally, in the last two sections of the results, we add the toxic effect. We assume that above a certain threshold, free toxin inhibits the bacterial growth. As noted by Klumpp *et al.* [26], a decrease in the growth rate will be reflected by a decrease in the transcription rates, therefore, we include the effect of the free toxin levels on the transcription rates as well.

### Similarities and differences in dynamics for three different TA modules

**Figure 2:**
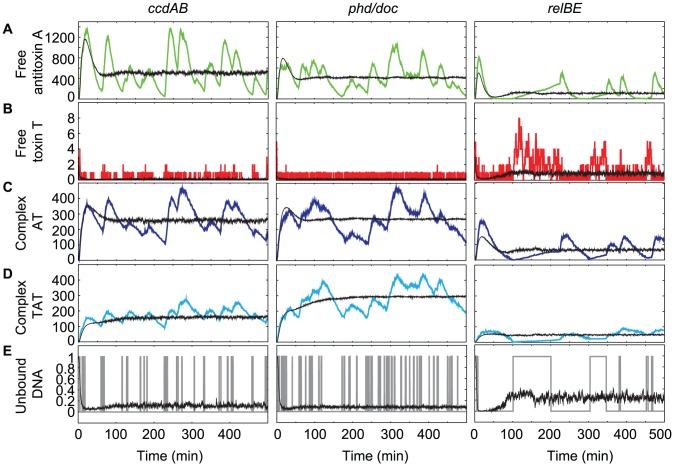
Numerical simulation of the generic TA model including two independent binding sites. The systems were simulated for 500 minutes per individual cell. The graphs show the results for a single cell (color-coded towards the species plotted: free antitoxin (green), free toxin (red), AT complex (dark blue), TAT complex (cyan) or free operator DNA (gray)) as well as an average of 1000 cells (in black). The used parameter sets are indicated above the panels. No toxic feedback effects are included: 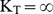.

In order to determine the influence of the parameter set used on the behavior of the toxin-antitoxin module, we performed stochastic simulations of a toxin-antitoxin module with two independent antitoxin binding sites on the promoter/operator, and the parameter sets for the *ccdAB*, *phd/doc* and *relBE* system (see Table 1 and Figure 2). Initially, mRNA is transcribed and toxin and antitoxin are translated (Figure 2A and B) as the operator DNA is initially unbound (Figure 2E). Once the operator gets bound, repression starts and mRNA transcription stops. From Figure 2E, it can be seen that after this initial response, the operator DNA is mostly occupied by one or more protein species. Pulses in the toxin and antitoxin level occur during short periods when the operator becomes unoccupied in a single cell. The free toxin population is retained at very low levels as is expected in a growing cell population [17]. For the *relBE* system, a maximum of eight free toxins is found for the simulated cell shown in Figure 2 and the average free toxin level is approximately one, whereas for the *ccdAB* and *phd/doc* systems, the average free toxin level is much lower than one (Figure 2B). The overall majority of toxin molecules are thus sequestered into toxin-antitoxin complexes AT (Figure 2C) or TAT (Figure 2D).

**Table 1:** Model Parameters for the *phd/doc*, *ccdAB* and *relBE* toxin-antitoxin systems.

Parameter	Meaning	*phd/doc*	*ccdAB*	*relBE*	Units
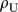	Unbound mRNA transcription rate	0.116086	0.1333	0.133	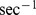
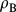	Bound mRNA transcription rate	0	0	0	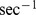
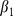	Antitoxin translation rate	0.137	0.139	0.127	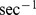
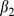	Toxin translation rate	0.053	0.033	0.02105	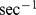
c	Translational coupling	3	3	10	
V	Volume factor	3.612e+8	3.612e+8	3.612e+8	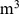
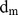	mRNA decay rate	0.00203	0.00203	0.00203	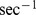
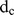	Decay rate due to cell cycle dilution	2.8881e-4	2.8881e-4	2.8881e-4	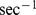
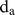	Antitoxin decay rate	4*dc	4*dc	0.00269	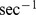
F	Decay of Antitoxin inside the complex	0.2	0.2	0.2	
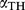	Binding of Antitoxin and Toxin through the high affinity site (T)	8.79e+6	2e+6	5.01e+5	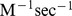
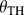	Unbinding of Antitoxin and Toxin through the high affinity site (T)	5.3e-5	7.14972e-6	1.66e-4	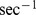
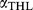	Formation of a bridge across two Antitoxins by one toxin using both a high and low affinity site (T)	8.79e+7	2e+7		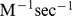
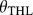	Unbinding of a bridge across two Antitoxins by one toxin using both a high and low affinity site (T)	2.74e-8	2.72e-11		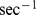
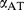	Binding of Complex (AT) to binding site on the operator	9625	3510	39000	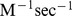
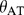	Unbinding of Complex (AT) from a binding site on the operator	0.0028875	0.001097	3.9e-4	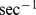
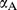	Binding of Antitoxin to a binding site on the operator	9625	3510	370	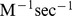
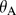	Unbinding of Antitoxin from a binding site on the operator	0.0231	0.008775	3.7e-3	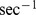
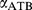	Binding of Complex (AT) to a binding site on the operator as well as formation of a toxin bridge with another antitoxin	96250	35100		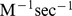
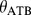	Unbinding of Complex (AT) from a binding site on the operator as well as unbinding of a toxin bridge with another antitoxin	1.49e-6	4.17e-8		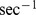
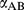	Binding of Antitoxin to a binding site on the operator as well as formation of a toxin bridge with a bound Complex (AT)	96250	35100		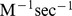
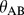	Unbinding of Antitoxin from a binding site on the operator as well as unbinding of a toxin bridge with a bound Complex (AT)	1.19e-5	3.33e-7		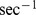

Although there are slight differences in the protein and complex concentrations and the number of binding/unbinding events on the DNA, the behavior of the toxin-antitoxin model with two independent binding sites on the operator is qualitatively similar for the *ccdAB* and the *phd/doc* parameter sets. The average levels are similar for the unbound DNA, antitoxin, toxin and complexes AT and TAT although the plotted single cell behavior differs due to the stochastic nature of the simulations.

The outcome of the simulations changes more significantly when the *relBE* parameter set is used. The most remarkable difference is the increase in the free toxin level for the *relBE* module. This is not illogical as the molecular mechanism for conditional cooperativity used by *relBE* is distinct from the mechanism employed by *ccdAB* and *phd/doc* and several parameters such as the DNA binding rates are very different. Considering the similarities in the output, for simplicity we only use the *ccdAB* parameter set in the simulations presented in the remainder of this work.

### Free toxin level is mainly controlled by complex sequestration and not by gene regulation

Two mechanisms are responsible for managing the potentially lethal toxin-antitoxin modules in bacteria. At the protein level, free toxins can be neutralized by complex formation with a free antitoxin or a non-saturated toxin-antitoxin complex AT [14]. At the transcriptional level, the negative autoregulation of the operon by conditional cooperativity ensures that the production of antitoxins and toxins is repressed when more antitoxin than toxin is present. When an excess of toxin emerges, the transcription is derepressed and the antitoxin will be the main product of translation, as explained above. To study the role of both levels in the regulation of toxin-antitoxin modules, we performed a series of simulations with the “independent binding sites” model, in which either operator binding or the sequestration of the toxin in complex TAT or in both non-toxic complexes (AT and TAT) were eliminated. When DNA binding by both antitoxin and toxin-antitoxin complexes is abolished during the simulation, the free toxin level remains fully controlled (see Figure S1). This shows that the sequestration into the complexes AT and TAT without any gene regulation accounts for a complete suppression of the toxin, albeit with a higher level for antitoxin and complexes AT and TAT. Alternatively, in simulations where formation of the secondary complex TAT (and therefore also conditional cooperativity) is eliminated, the cell continues to control the toxin level, although more variability in the antitoxin level is observed. When the formation of both complexes AT and TAT is abolished, but DNA binding remains included, the cell does not manage to control the free toxin level and produces as much toxin as antitoxin. This suggests that AT formation is necessary for the control of the free toxin level and TAT formation helps to reduce the variability in the antitoxin level.

### An increase in the number of binding sites on the operator allows for toxin control with lower protein levels and leads to a localized response in time

**Figure 3:**
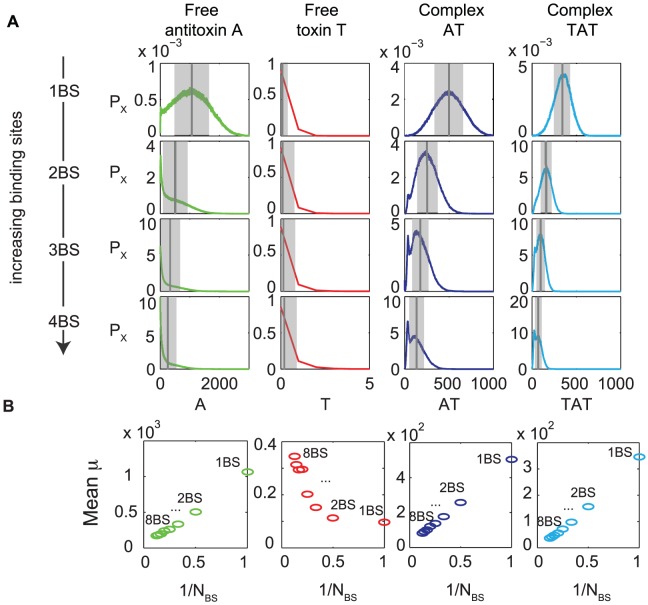
The levels of antitoxin and toxin-antitoxin complexes decrease with increasing number of operator binding sites. (A) Probability density functions (p.d.f) for free antitoxin, free toxin, free complex AT and free complex TAT using the generic model with independent binding sites on the operator are shown. 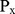 refers to the probability of finding the species 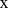 (where 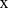 can be 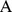, 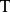, 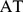 or 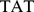) with a given amplitude. The gray-shaded region shows the margin of error with width 2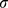, the dark gray line shows the mean. (B) Mean value of protein level versus the reciprocal number of binding sites on the operator (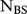). No toxic feedback effects are included: 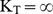.

Different toxin-antitoxin modules have different numbers of binding sites on their operator, ranging from two in the *phd/doc* and *relBE* system [20], [33] to eight in the *ccdAB* system [34]. We investigated the influence of this property on the levels of free toxin, free antitoxin and non-toxic complexes in a toxin-antitoxin system with independent binding sites on the operator. In Figure 3A, we plot the Probability Density Functions (p.d.f) for each of the protein components of the toxin-antitoxin system. The p.d.f. is constructed by simulating the time evolution of many cells and detecting the protein level at each point in time. Using this information we calculated the probability to find a certain number of protein components in a cell. It can clearly be seen that increasing the number of binding sites on the operator leads to decreased protein levels and variability for the free antitoxin and complexes (AT and TAT), while the free toxin level stays low and relatively constant. This decrease in the protein concentrations allows a more economical maintenance of the toxin-antitoxin system. The increase from one to two binding sites on the operator has the most profound effect on the protein levels. The mean value for each distribution is shown in Figure 3B and there is a linear relationship with the reciprocal of the number of binding sites (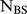) on the operator for A, AT and TAT. There seems to be a direct correlation between the free toxin variability and the number of binding sites, however the absolute magnitude of this phenomenon in comparison to the total amount of antitoxin and complexes makes this relationship negligible.

The p.d.f. for both of the non-toxic complexes can be described by a normal distribution for a toxin-antitoxin system with one binding site on the operator. However, with an increasing number of binding sites these distributions become bimodal. The extra peak at low complex concentrations may be explained by the fact that the antitoxin level equals zero more often as the number of binding sites on the operator increases (see Figure S2 and S3).

The effect of the number of independent binding sites on the operator on the time evolution of the antitoxin and toxin level and the binding on the DNA is shown in more detail in Figure S2 and S3. When the operator only consists of one binding site, many fast DNA binding and unbinding events are observed. This leads to an evenly distributed response around the average for the mRNA production and therefore the free toxin and antitoxin level. With an increasing number of antitoxin binding sites on the operator, the probability of the operator being bound by at least one antitoxin increases as well. This leads to localized bursts in time of mRNA creation and corresponding spikes in the free toxin and antitoxin levels.

### Conditional cooperativity is essential to maintain a viable toxin∶antitoxin ratio in TA modules with interacting binding sites on the operator

Conditional cooperativity is included in both the model for independent binding sites and the model with interacting binding sites, since the toxin can derepress the operon at high 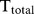
∶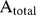 ratios. In the former model, this is due to the assumption that a TAT complex is unable to bind the DNA. In the latter model, this is due to the fact that “stripping” of a protein chain from the promoter/operator can occur when a low affinity interaction in this chain is replaced by a high affinity interaction with a new toxin, forming soluble TAT complexes that are unable to occupy adjacent binding sites on the operator due to steric hindrance. The role of conditional cooperativity in the regulation of TA modules is studied in the following simulations by abolishing the formation of TAT complexes on the DNA and their subsequent release (independent binding sites) or the stripping reaction (interacting binding sites). In both cases, the formation of TAT complexes in solution is still possible.

**Figure 4:**
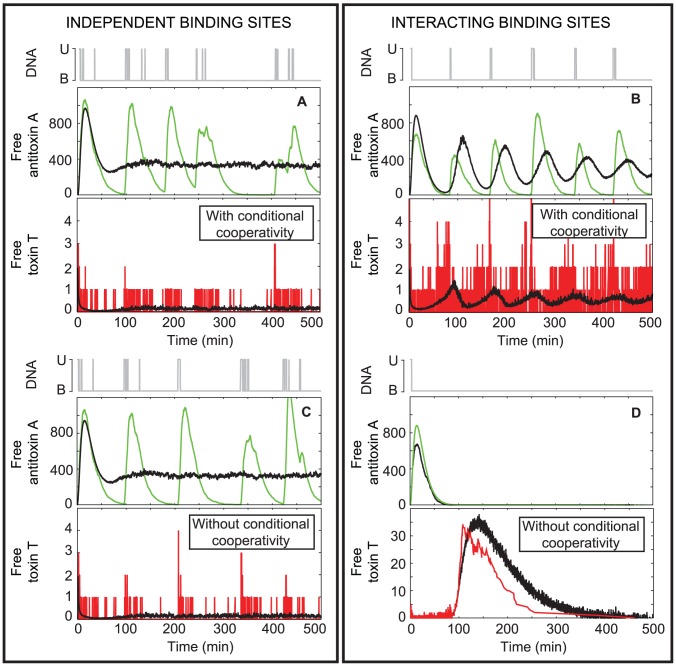
Conditional cooperativity has a larger influence on system dynamics in the interacting binding sites model. The graphs show the time evolution of a single cell (color-coded towards the species plotted: free operator (gray), free antitoxin (green), free toxin (red)) as well as an average of 1000 cells (in black). A and C: three independent binding sites on the operator. B and D: Three interacting binding sites on the operator. A and B: with conditional cooperativity. C and D: without conditional cooperativity. No toxic feedback effects are included: 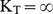.

When the operator consists of independent binding sites (Figure 4A and C), the unbinding rates of antitoxin and AT from the operator are large enough to free the promoter and allow mRNA creation. Therefore, conditional cooperativity has no profound effect on the system dynamics as the DNA binding reaction rates control the behavior of the toxin-antitoxin system.

In the model with interacting binding sites (Figure 4B and D), however, conditional cooperativity is of essential importance to free the DNA promoter/operator from the chain of alternating toxins and antitoxins bound to it, so that transcription can occur and the antitoxin can be expressed (Figure 4B). In this case, the toxin level can be controlled. In the absence of conditional cooperativity, the promoter/operator remains bound as the unbinding rates are too slow to completely free the DNA from the protein chain. In this situation, no toxin or antitoxin is expressed. As the antitoxin will be degraded more rapidly, a large increase in the free toxin level occurs, inducing a cessation of cell growth or cell death (Figure 4D). Please note that the decrease in the toxin level after this spike is not necessarily found *in vivo*. This decrease is caused by toxin dilution due to cell division, as we assumed that the doubling time of *E. coli* is constant. Furthermore, the synchrony in the average antitoxin and toxin concentrations in Figure 4B is caused by the initial conditions being identical for all cells. These coherent oscillations disappear after longer simulation times, but reflect the presence of a well-defined time between spikes in the free toxin and antitoxin level. Such coherence is not likely to be found in an actual bacterial population though due to the lack of similar synchronous initiation of the different cells.

### Free toxin accumulates when the toxin translation rate exceeds twice the antitoxin translation rate

**Figure 5:**
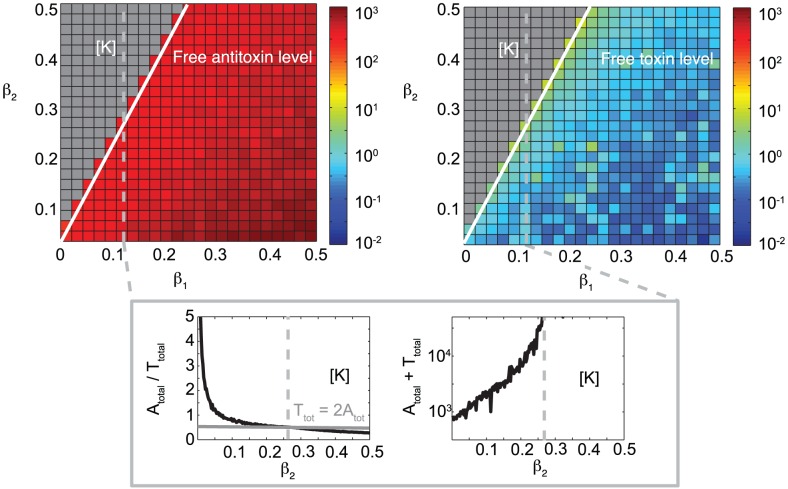
If the toxin translation rate exceeds twice the antitoxin translation rate, free toxin accumulates. Parameter scans for 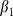 versus 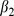 show the level of the free antitoxin A and the free toxin in the case of independent binding sites on the operator. The response of 200 cells has been averaged after simulating for 500 minutes. In the region [K], indicated in gray, the free toxin level grows continuously to large numbers, corresponding to a non-culturable cell population. The lower panels show the effect of a change in 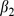, keeping 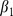 at its normal value (see table 1), on the 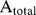
∶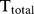 ratio and total protein number (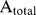
+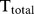). No toxic feedback effects are included: 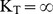.

The translation rates for the antitoxin and the toxin, 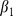 and 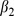, are hard to determine experimentally but are important parameters for the behavior of the toxin-antitoxin module. The translation rates in this article are based on the average translation rate in *E. coli*, on the lengths of the proteins, on the fact if monomers or dimers are formed in solution (immediate dimerization is assumed and therefore the translation rate is halved in the case of dimers) and on the translational coupling, ensuring that toxins are produced at a lower rate than antitoxins. In order to show the influence of variations in both translation rates, Figure 5 shows the free antitoxin and the free toxin level in the parameter plane (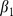, 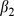), using the model for independent binding sites on the operator. Two regions are clearly visible in this parameter space: One in which the free antitoxin level is high and the free toxin level is controlled (on average less than one free toxin per cell is present) and one in which the free toxin level is very high with negligible amounts of free antitoxin present, corresponding to a non-culturable cell population. The latter region is indicated as [K] in Figure 5. There is a clear threshold between these two cell populations, which is crossed when the toxin translation rate exceeds twice the antitoxin translation rate. In all currently investigated TA modules, the synthesis rate for the antitoxin was higher than the one for the toxin [17]. Therefore, these modules can be safely maintained in a cell population.

The lower panels in Figure 5 show the effect of 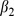 on the 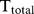
∶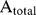 ratio, and on the total protein number (keeping 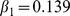 as in all previous simulations). From these plots it can be seen that in controlled, stable cells the total amount of toxin is always lower than twice the total amount of antitoxin. This can be explained by the fact that one antitoxin can maximally neutralize two toxins for the investigated TA modules [25], [34], [35]. At the boundary 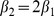, the critical 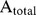
∶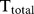 value of 0.5 is reached. If the toxin translation rate is further increased, the total level of toxin is larger than twice the antitoxin level and free toxins can accumulate. When approaching the 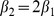 boundary, the total protein level in the cell also becomes increasingly large. This boundary can be found analytically from the deterministic version of the “independent binding sites” model under certain assumptions (see Text S1).

### TA modules allow for rare, extreme stochastic spikes in the toxin level

**Figure 6:**
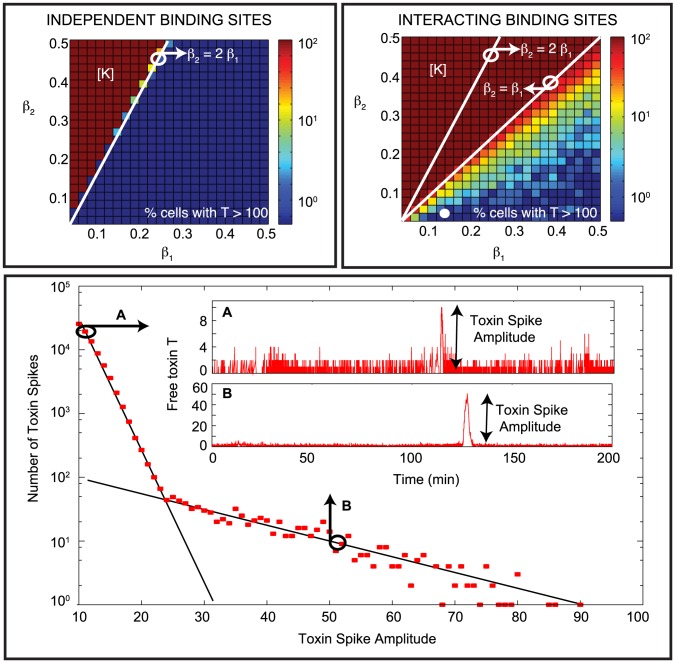
The antitoxin and toxin translation rates influence persister cell formation. The parameter scans for 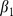 versus 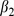 in the top panels show the percentage of cells (out of 200 simulated cells) that reach a free toxin level higher than 100 during a time of 500 minutes, both for independent and interacting binding sites. The bottom panel provides a more detailed analysis of the behavior observed in the interacting binding sites model for the parameters presented in Table 1 (see also white dot in the parameter scan). The number of toxin spikes with amplitude larger than 10, detected by analyzing the time evolution of 320000 cells during a time of 500 minutes each cell are plotted. Two characteristic scaling laws are found. The first one (A) is related to regular stochastic variation, and a second one with lower probability is related to rare events where a TAT complex stays bound on the DNA for a limited time determined by the DNA binding affinity of TAT. Panel A shows an example of a typical time series of 200 minutes of regular cell dynamics. Panel B shows a similar time series containing a rare toxin spike of high amplitude, which is a potential avenue to persistence. No toxic feedback effects are included: 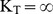.

TA modules are involved in the emergence of persister cells [14], [30], [31]. In the following paragraphs, we check which parameters and assumptions are necessary to allow a persister to be formed, and reveal one possible avenue to persistence. A parameter scan in the translation rate of the antitoxin and the toxin was performed, both for the model with three independent binding sites on the operator as for the model with three interacting binding sites. The top panels in Figure 6 show the percentage of cells that reach a toxin level higher than 100 during a time interval of 500 minutes. In accordance with Figure 5, a sharp transition from 0% to 100% can be observed when the translation rate of toxin 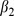 exceeds twice the translation rate of antitoxin 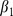 for the independent binding site model. Below this boundary, the toxin level is controlled in every cell (see for example Figure 4A). When crossing this boundary, the toxin level continuously grows to large values (see Figure S5B). Of course, the *in vivo* response may differ from the shown simulation once the toxin level reaches a sufficiently high level, as the toxic effect is not explicitly modeled in this simulation. However, as high toxin levels would be present in every cell, growth of a bacterial cell population would be impossible in this region of parameter space as indicated above (Figure 6 region [K]).

In the case of three interacting binding sites on the operator, extra effects come into play. For 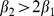 every cell still experiences a continuously growing toxin level (see Figure S5B). However, in the experimentally most relevant case, where the translation rate of toxin is smaller than the translation rate of antitoxin [17], the observed response differs from cell to cell. In this region, two types of response are possible with different probabilities. The cell can have a stable low toxin level, controlled by regular oscillations in the antitoxin level (see Figure 4B and Figure 6A). In this case, each increase in the toxin∶antitoxin ratio is followed by the release of the protein chain from the DNA, causing a spike in the mRNA, antitoxin and complex levels, respectively, and keeping the free toxin level close to zero. This response is similar to the one in the case of independent binding sites on the operator.

The other possible response is that the cell produces a large pulse of toxin (see Figure 6B). The toxin level does not continuously grow, but its growth is arrested after some time. However this is abated since after this occurrence the system quickly returns to its controlled state, because no toxic effect was included in this simulation. This rare event can be stochastically initiated if a TAT complex is still bound on the DNA when the 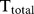
∶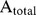 ratio reaches the level of two, this is when the level of the antitoxin and the AT complex are very low or zero. In this case, the full chain of alternating toxin and antitoxin dimers can no longer be formed on the DNA. As conditional cooperativity is unable to free this complex from the DNA, the free toxin level will rise as long as the TAT complex does not unbind from the DNA. Please note that this rise in free toxin level is caused by the degradation of the antitoxin within the complexes AT and TAT, and the concomitant release of toxin molecules. Therefore, degradation of antitoxin within the toxin-antitoxin complexes is necessary to obtain persister cells in this framework.

The probability of having toxin spikes, and therefore the potential of persisters occurring in the population, increases as one approaches the 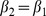 line. The toxin spike becomes increasingly high with increasing values of 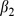 (see also Figure S4). In the region 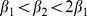 every cell will reach toxin levels higher than 100, but the response can either be a toxin spike or a continuously growing toxin level (see Figure S5A and B respectively). The percentage of the cells responding with continuously growing toxin levels increases (to 100%) as one approaches the 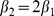 line.

The bottom panel of Figure 6 shows a more detailed analysis of the probability of obtaining large amplitude toxin spikes for the normal parameter values as presented in Table 1. The number of toxin spikes having an amplitude larger than 10 is numerically detected. One observes two characteristic scales. The first one is associated to regular stochastic fluctuations of the toxin amplitude under normal operation (see Figure 4B and Figure 6A). The probability of finding toxin spikes of increasingly high amplitude decreases exponentially. The second scaling can be attributed to the different mechanism where a TAT complex remains bound to the DNA for a certain time, as mentioned above. Provided that the binding affinity of TAT to the DNA operator site and the toxin translation rate 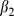 are large enough, rare high amplitude toxin spikes can be observed (see also Figure S4).

### Large toxin spikes provide a route to persister cell formation through growth rate suppression

To obtain a more realistic view of persister cell formation, the duration of persistence and the influence of free toxin levels on the growth rates, we introduced toxic feedback effects into both the independent and interacting binding site models. Once the free toxin level crosses a threshold 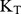, the growth rate and transcription rates decrease. This decrease is modeled by a Hill function where the Hill factor 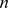 determines how sharp the transition is around the threshold value 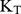. A minimal growth rate is defined to ensure that the cell can always recover after a (potentially long) time (see also Materials and methods).

In the independent binding site model toxic feedback effects have only marginal impact and no long-term persister dynamics are found (see Figure S6). Panels A and B in Figure S6 show a direct comparison between simulations carried out without feedback (see also Figure 3) and with feedback. Even in the case of a very low threshold, the protein levels remain very similar and almost no difference is observed in the p.d.f. Panel C in Figure S6 shows the effect of the threshold level chosen and its impact on the normalized individual fitness or growth rate 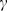 (where 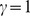 in the case of no growth rate reduction). The fitness is detected at each point in time of the simulation and used to calculate its probability 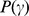 at any given time. At high threshold values, free toxin levels remain too low to be able to cause a noticeable reduction in fitness. When decreasing the threshold, the fitness landscape is broadened due to stochastic excursions of the free toxin level, allowing for lower growth rates. We have calculated the average fitness R by taking the first moment of the probability distribution of the individual fitness (R = 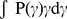) and it is displayed in the legend. Although a modulation of the growth rate can be obtained, at no point is the dynamics altered and no clear switch to a persister state is observed.

**Figure 7:**
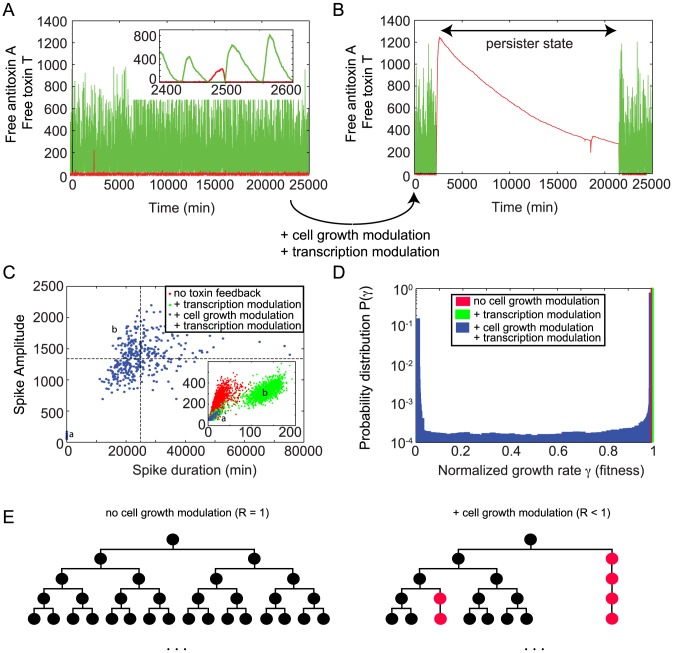
Large toxin spikes provide a route to persister cell formation through growth rate suppression. Panel A and B show the free toxin T (red) and free antitoxin A (green) level in the case of interacting binding sites on the operator, respectively without (A) and with (B) toxic feedback effects. Panel C shows a scatterplot comparing toxin spike amplitude and duration when the system has no toxin feedback (red), transcription modulation only (green) and transcription and cell growth modulation (blue). Panel D shows a probability distribution of the fitness landscape in the three cases, obtained by analyzing the response of 100 cells during 100 days of simulated data. Panel E shows a sketch of how the average growth rate of a population of cells can be decreased through the presence of persister cells (cells with decreased growth rate). Such persister cells are shown in red and their growth is largely arrested. 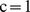, 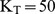, 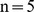 and 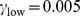.

The simulations using the interacting binding site model with toxic feedback effects are shown in Figure 7, where we have decreased the translational coupling by a factor of three (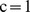) such that toxin spikes are more likely to be found. Panels A and B show a simulation without and with the inclusion of toxic feedback effects, respectively. When no feedback is included the system responds to the toxin spike by complex sequestration that causes a return to nominal levels of toxin. However, with feedback a toxin spike of significant size can cause the system to switch to a persistent state for multiple cell cycles where there is no antitoxin present to neutralize the toxin levels. The duration of this persister state is closely related to the spike amplitude, as the recovery time to switch back to normal operation is mainly determined by the time it takes for the toxin level to drop due to (slow) dilution. This close relation between toxin spike amplitude and duration is shown in Panel C. Without toxic feedback the red cluster of points shows a clear correlation between spike amplitude and duration (see inset). When only introducing a toxic effect on the transcription rates, this cluster of points is split in two separate ones (see clusters a and b in green). If one also introduces a toxin-dependent growth rate modulation (see blue points), cluster (a) remains similar, but the second cloud of points (b) shifts to duration times that are orders of magnitude larger. This is immediately reflected in the fitness landscape shown in Panel D for the three different cases. Including cell growth modulation, one can now observe that it is most probable to find the cell in a state with fitness 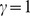. However, there is a clear second peak in the probability distribution at a much reduced fitness 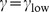. The shape of this bimodal distribution function (such as for the relative heights of both peaks) can be controlled by changing the various system parameters. Similarly the average fitness R can be controlled. The bimodal response is qualitatively very different from the case in the independent binding site model and originates from the possibility to create the persister states (where the fitness can be decreased for longer periods of time). Similar bimodal effects have been studied in other papers [26], [29], [36]. However, no bistability is present in our model when including the toxic feedback, provided the minimal fitness 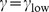 is non-zero. The system remains monostable, but the bimodal response results from stochastically triggered transient excursions during which the individual fitness is very low.

Panel E shows a sketch of normal exponential cell population growth (left) and a reduced growth of a cell population due to persister cell creation (right). The average fitness of a population of cells can be decreased through the presence of persister cells which are highlighted in red. Since these cells have their growth arrested at points they do not divide on the usual time scale as the normal (black) cells. This is why the population which has these persisters (right) can have a lower population number or slower growth in comparison to a population without persister cells (left).

### Persistence can be greatly increased during nutritional stress

During nutritional stress, the antitoxin degradation rate 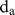 increases due to the activation of cellular proteases like Lon [37]. Furthermore, the rate of protein synthesis decreases to approximately 5% of the pre-starvation level [38]. We thus investigated the influence of the antitoxin degradation rate and the antitoxin and toxin translation rates (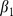 and 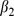) on the free toxin level in the independent binding sites model (see Figure S7). It can be observed that the boundary 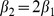 for the viability of a cell population does not change when increasing the antitoxin degradation rate. For the viable cells, the increase in the antitoxin degradation rate is mainly responsible for the increase in the average free toxin level associated with nutritional stress [38]. A decrease of both the toxin and antitoxin translation level with the same factor will not heavily affect the free toxin level.

We further investigated if the increase in antitoxin degradation and the decrease in translation rates and in growth rates associated with nutritional stress also affect the formation of persister cells. In Ref. [12], Balaban *et al.* outlined a model for persisters created through normal growth (type II) and showed a switch from normal behavior to persistent activity in a population. The model has two states, normal (N) and persister (P), the switching rate from N to P and P to N are defined as a and b, respectively, while the growth rate of both states are given by 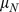 and 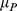:
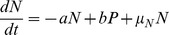
(1)

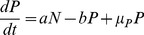
(2)


**Figure 8:**
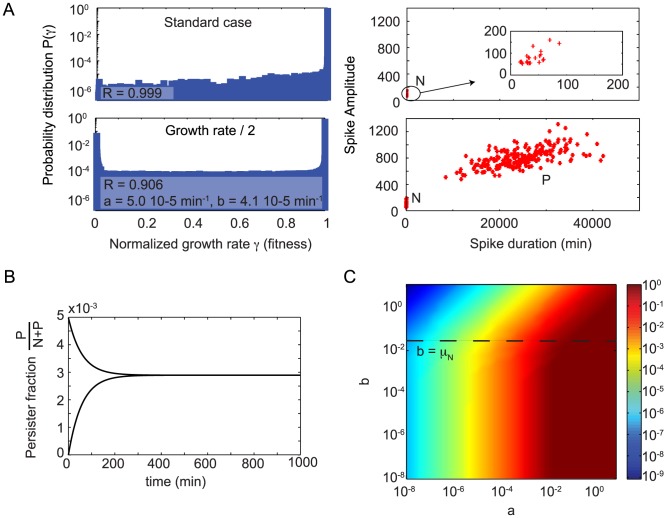
Growth rate modulation can cause an increase in the amount of persisters during nutritional stress. Panel A describes the probability distribution of the fitness landscapes (left) and the correlation between spike duration and spike amplitude (right) for the normal parameter set and the parameter set in which the growth rate is halved, in the model with interacting binding sites on the operator. The corresponding normalized average growth rate R and transfer rates from normal to persister state are shown. 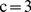, 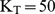, 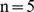 and 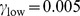. Panel B shows the time evolution of the persister fraction, starting from two different initial conditions, using 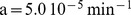 and 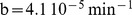. In panel C, parameter scans for a versus b show the persister fraction.

The same model can be used to analyze the growth of cell populations in our case. The mentioned switching rates a and b can be directly estimated from our simulations and changes in these rates can be linked to underlying system parameters and the corresponding dynamics. Both growth rates 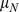 and 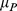 correspond to both peaks in the bimodal fitness distribution (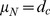, 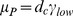). We used our model to analyze the fitness landscapes and estimate corresponding switching rates resulting from the changes in growth, translation and antitoxin degradation rates. In the top panels of Figure 8A, using the standard parameter set, no well-separated persister population is found in the scatterplot showing the toxin spike amplitude vs. the spike duration. This absence of a clear family of persister cells is also reflected in the fitness landscape where no bimodal response is found. However, when the growth rate is halved, there are two distinct populations of cells (see bottom panels A). In addition to the population dividing at a normal growth rate, there is a fraction dividing at a fitness 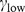. The estimated switching rates show that the transfer from normal to persister state occurs at a faster speed (a>b) than the return. This difference in speed becomes more pronounced when also including a reduction in translation rate and antitoxin degradation rate (see Figure S8).

Using Eqs. (1)–(2), the time evolution of both cell populations can be simulated in time. The resulting persister fraction is shown in Figure 8B for the switching rates as estimated from the case with halved growth rate. Independently from the initial conditions, the persister cell fraction evolves to a steady state solution after about 300 minutes. A similar two-state population dynamics model has been used in Ref. [29] to understand how the combination of cellular memory and individual fitness jointly define the overall distribution of cell populations. In this work, phenotypic switching rates were estimated in a bistable system of high and low expressers, and it was shown how cell lineage statistics can be different from population snapshot statistics. The authors concluded that cells tend to switch predominantly to the high expression state and switch back much more rarely. This translates to a>b, which agrees with our findings in the presence of nutritional stress. An example of this behavior can be seen in Figure 7B, where a typical time series is shown of an individual cell lineage. It is clear that the cell spends most of its time in the persister state. Looking at the persister fraction of the overall cell population, however, only a minority of the cells are in a persister state.

The persister fraction in the overall cell population is greatly determined by the switching rates to get into a persister state and to escape from it. This escape time is essentially determined by the reduced fitness in such a persister state. Although we have found that during nutritional stress a>b, the normal cell population still dominates due to its much larger individual fitness with respect to the persisters. Figure 8C shows an analysis of the dependence of the persister fraction on both switching rates a and b. One can clearly see that the switching rate to get into persistence strongly controls the persister fractions, such that its increase in nutritional stress conditions immediately leads to an increased persister fraction. The return rate to normal operation (b) has practically no influence on the persister fraction, provided that it is slower than the decay rate due to dilution of the normal cell population (related to its growth rate).

## Discussion

Strong evidence has been accumulating that various types of bacterial toxin-antitoxin modules are implicated in persister cell formation [14], [30], [31]. In the present paper we investigated how the peculiar type of gene regulation called “conditional cooperativity”, that seems to be a common feature of TA modules, is capable of controlling the cellular free toxin levels and might control the formation of persisters. We successfully constructed two models for the autoregulation of toxin-antitoxin modules by conditional cooperativity, which mirror two molecular mechanisms that allow for conditional cooperativity [19], [23], [25]. In the first model, we consider the binding sites on the operator as independent entities on which antitoxins and AT complexes can bind, whereas in the second model, the toxins can bridge the antitoxin-bound binding sites on the DNA. Stochastic simulations based upon these models showed several essential characteristics of TA modules, such as very low free toxin levels and high free antitoxin levels in non-starvation conditions, and this for three different parameter sets derived from experimental data available for F-plasmid *ccdAB*, bacteriophage P1 *phd/doc* and *E. coli relBE*.

We found that sequestration of toxins in toxin-antitoxin complexes and not gene regulation is responsible for the main control of the free toxin level as a viable toxin-antitoxin balance is still maintained in absence of any regulation (removing the DNA binding properties of the antitoxin from the model). However, when the DNA binding reactions are included in the “interacting binding site” model, the “stripping” reaction (binding of T to AT to obtain a TAT species that quickly dissociates from the operator) is still necessary to allow fresh antitoxin synthesis and therefore maintain viable free toxin levels. The stripping reaction, which has a pivotal role in the conditional cooperativity, allows the toxin to function as a derepressor for the operon by releasing the chain of alternating toxins and antitoxins from the DNA at high 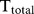
∶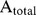 ratios. In the independent binding site model, such a chain cannot be formed. Therefore, the dissociation rates of the antitoxin and the AT complex from the DNA are sufficiently high to free the operator, allowing antitoxin synthesis and subsequently toxin neutralization.

We further found that the toxin level can be controlled in the presence of lower amounts of antitoxin and toxin-antitoxin complexes if the number of binding sites on the DNA increases. Therefore, the maintenance of a TA module becomes more economical for the cell as the amount of binding sites on the operator increases. This may be the reason why the *ccdAB* module has evolved to have as much as eight binding sites on the operator.

When considering independent binding sites on the operator, parameter scans reveal a clear threshold between healthy, antitoxin dominated, and non-culturable, toxin dominated cell populations, which is crossed when the toxin translation rate is more than double the antitoxin translation rate. In the model with interacting binding sites on the operator, toxin accumulation also occurs in all cells above this boundary. In all studied TA modules, the antitoxin translation rates are higher than the toxin translation rates [17]. In this region in parameter space, most cells have a low free toxin level, but in very rare cases the free toxin level spikes, which can lead to the formation of a persister cell. This steep increase in the free toxin level can occur when the operator is occupied and no new antitoxin can be made at a moment when the free antitoxin level is very low. In this case, the degradation of antitoxin in toxin-antitoxin complexes leads to the accumulation of free toxins, which can perform their toxic activity. This toxic activity is added in certain simulations by decreasing the growth rate and the transcription rate once the free toxin concentration exceeds a certain threshold. In this case, the level of free toxin determines how long the toxin spike lasts and how long the cell resides in the persister state. A similar result was obtained by Rotem *et al.*, who found that bacteria go into a dormant state once the toxin level crosses a threshold, and that this toxin level determines the length of the dormancy [36]. In reality, more complex toxic feedback effects can also take place, dependent on the TA module considered. For example, in the case of RelE or Doc, translation would be inhibited *in vivo*. As multiple TA modules can be present in one bacterium, the inhibition of translation by one toxin could lead to an increase in the concentration of other toxins as suggested by Keren *et al.* [14].

In order to obtain persister cells during our simulations, it was necessary to assume that antitoxins can be degraded within the toxin-antitoxin complexes. It was also previously shown that such degradation can play an important role in TA modules, as a switch from an antitoxin dominated state to a toxin dominated state upon amino acid starvation was only possible for the *relBE* system when the active degradation of RelB within toxin-antitoxin complexes was taken into account [32]. Moreover, we found that the increase in the amount of persisters during starvation is mainly caused by the increase in the antitoxin degradation rate and the decrease in the growth rate, rather than by the decrease in the translation rates of the toxin and the antitoxin.

As toxin-antitoxin modules are very complex systems, even more interactions could be integrated in the models. For example, it would be interesting to examine the influence of the mechanism for the toxicity on the dynamics of a TA module. This mechanism is specific for every toxin-antitoxin module, for example mRNA degradation in the *relBE* TA module and inhibition of translation in *phd/doc*. Our model also assumes that the operator of a TA module consists of binding sites with identical affinity. It will be of interest to investigate the dynamics of a TA module with an operator that contains several binding sites with different affinities for the antitoxin.

Finally, we would like to develop a more general interacting binding site model, removing the need of simulating all DNA interactions separately. Such a model would allow a more in-depth investigation of the dynamical mechanism leading to the described rare toxin spikes. So far, it seems that these spikes are triggered stochastically and do not exist in the deterministic system, being always monostable. In most systems where pulsed dynamics have been observed, however, they often rely on underlying deterministic bifurcations leading to for instance bistability, oscillations and excitability (for an overview, see Ref. [39]). One such example is for instance the genetic competence in *Bacillus subtilis* under stress conditions, where a transient cellular state is also initiated stochastically [40].

## Materials and Methods

### Model parameters

Three parameter sets were built up, one for the *phd/doc*, one for the *ccdAB* and one for the *relBE* toxin-antitoxin module (Table 1). mRNA transcription is assumed to only take place when the promoter/operator region is unbound, hence 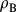, the transcription rate for bound DNA, is zero. The transcription rate for unbound DNA, 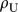, is based on a transcription rate of 70 nucleotides/second [41], [42] and the transcript lengths. The translation rates are based on a translation rate of 20 amino acids per second [43]. Furthermore, the parameter set accounts for the fact that CcdA, CcdB, Phd and RelB form dimers in solution, whereas Doc and RelE are monomers. The *in vivo* translation rates for the antitoxins (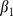) are higher than the ones for the toxins due to translational coupling. As such, in order to evaluate the translation rate for the toxin (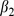), the translation rate based on the length was divided by the translational coupling factor (c). The volume factor (V) allows us to convert molar units to molecules/cell, using an *E. coli* volume of 0.6 (μm)^3^ [44].

The decay rate of the mRNA (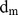) is based on a half life of 5.7 minutes *in vivo*. Cell division is not explicitly included in the model, but it is implicitly present in the decay rate 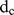 for the toxin and the complexes AT and TAT. These values were chosen so that the amount of proteins in the cell is halved every generation and the doubling time of *E. coli* was set at 40 minutes. As the antitoxins are always degraded faster than the corresponding toxins, the antitoxin decay rate 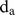 was fixed as four times 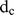, corresponding to a half life of approximately 15 minutes for CcdA [16]. We include antitoxin degradation in AT and TAT complexes. This is described by the parameter F, which is set at a certain percentage of 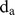.

Both in the *ccdAB* and in the *phd/doc* system, the antitoxin can bind to a high affinity and a low affinity binding site on the toxin. The 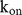 for the interaction of CcdA at the high affinity binding site, 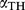, was determined by Surface Plasmon Resonance (SPR) by De Jonge *et al.* [45]. The 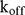 (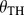) for this interaction is calculated from this 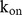 and the 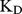 for the high affinity toxin-antitoxin interaction, determined by Drobnak *et al.* [46] using ITC. These kinetic parameters are based on SPR results (Loris and Garcia-Pino, unpublished data) for the *phd/doc* operon and on SPR results by Overgaard *et al.* [47] for the *relBE* operon.

The 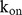 for the interaction of CcdA with one binding site on the operator, 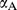, was determined as 3510 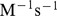 (Loris *et al.*, unpublished data); the 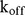 for this interaction, 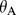, is based on a 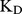 of 2.5 μM [48], and 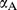. For the *phd/doc* operon, the 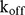 of the antitoxin from the DNA is based on a half life of 30 seconds for a complex of Phd and a single binding site on the operator [49]. The 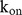 for this interaction is based on the 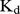, determined by Garcia-Pino *et al.* [23] using ITC and this 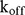. It was assumed that the 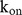 for a toxin-antitoxin complex (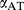) is equal to the 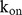 for an antitoxin alone. The higher affinity of this complex for the operator DNA, derived from EMSA experiments, is therefore reflected in the 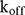 (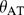) alone.

For the *relBE* operon, the dissociation rates of the antitoxin and the toxin-antitoxin complex AT from the DNA were determined by Overgaard *et al.* [20] using SPR, while the corresponding association rates are based on the dissociation constants reported [50]. The 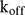 of a TAT and an AT complex from the DNA are assumed to be equal in the model with interacting binding sites on the operator, whereas the TAT complex immediately unbinds in the independent binding sites model. When one protein or protein complex interacts at two different sites with proteins or DNA with known affinity, for example when a toxin forms a bridge between two bound antitoxin molecules by binding one antitoxin at the high affinity and one antitoxin at the low affinity binding site, these affinities are multiplied. We assume the 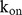 for the binding of all proteins and complexes to the DNA or to a DNA-bound protein complex to be equal to the 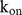 of the antitoxin to DNA, unless a supplementary high affinity toxin-antitoxin interaction is formed in the process. In this case, the 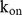 is multiplied by ten.

In certain simulations, we introduce toxic feedback effects (see also Figure 1). Firstly, we describe a decrease in transcription rate as a function of the free toxin level:
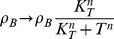



Secondly, we consider the decrease in the growth rate (modeled by an equivalent decrease in the dilution rate 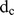) as a function of the free toxin level:
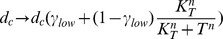



Each of these effects is implemented by a Hill-type function, with 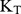 the toxin threshold, n the Hill factor describing how sharp the transition takes place around 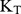 and 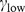 defined as the lowest possible normalized growth rate at very high levels of free Toxin T. We define 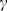, the normalized growth rate, as:
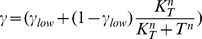



We use 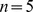 and 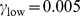 and unless otherwise stated, we use 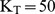. In order to obtain a high number of persisters, we decreased the translational coupling, 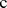, to 1 instead of 3 in Figure 7.

### Gillespie algorithm

The outlined models were simulated using a Gillespie algorithm which is based on treating the chemical reactions as discrete stochastic events [51]. At each time step, the state of the system is given by the number of molecules (or equivalently: the concentration) of mRNA (M), antitoxin (A), toxin (T), primary complex (AT) and secondary complex (TAT). The operator was defined as having n binding sites, denoted by 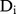, being 0 if bound and 1 if unbound. The total operator site is unbound if 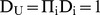 and bound if 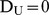.

The chemical reactions at the protein level with the rates determined by the parameters specified in Table 1 lead to the changes in the number of molecules as outlined in Table 2.

**Table 2:** Core reactions for the Independent and Interacting binding site models for the Gillespie simulation.

Protein Level Reaction	Propensity	M	A	T	AT	TAT
Bound mRNA Creation	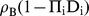	+1	0	0	0	0
Unbound mRNA Creation	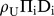	+1	0	0	0	0
Decay mRNA	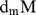	−1	0	0	0	0
Antitoxin Creation	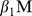	0	+1	0	0	0
Decay Antitoxin	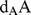	0	−1	0	0	0
Toxin Creation	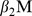	0	0	+1	0	0
Decay Toxin	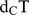	0	0	−1	0	0
Complex (AT) Creation	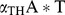	0	−1	−1	+1	0
Breakdown of Complex (AT)	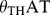	0	+1	+1	−1	0
Decay Complex (AT)	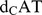	0	0	0	−1	0
Decay of A within Complex (AT)	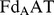	0	0	+1	−1	0
Complex (TAT) Creation	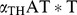	0	0	−1	−1	+1
Breakdown of Complex (TAT)	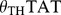	0	0	+1	+1	−1
Decay Complex (TAT)	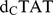	0	0	0	0	−1
Decay of A within Complex (TAT)	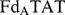	0	0	+2	0	−1

M: mRNA, A: free antitoxin, T: free toxin, AT and TAT: toxin-antitoxin complexes. The meaning of the model parameters is given in Table 1.

## Supporting Information

Figure S1 **Sequestration in complexes, rather than gene regulation, controls the free toxin levels.** The panels on the left hand side show the single cell response, while the panels on the right hand side show the average response of 1000 cells. The black line is a normal simulation using the stated parameters. The green dotted line is the simulation result without any protein binding to the DNA promoter/operator site. The red line excludes sequestration of toxin into the complex TAT, while the light blue line excludes sequestration of toxin in any complex (both AT and TAT).
(EPS)

Figure S2 **As the number of binding sites on the operator increases, the response becomes more localized in time.** The systems were simulated for 500 minutes per individual cell. The graphs show the time evolution of a single cell (color-coded towards the species plotted: free operator (gray), free antitoxin (green), free toxin (red)) as well as an average of 1000 cells (in black).
(EPS)

Figure S3 **Regular oscillatory behavior is observed in the model with interacting binding sites.** Time evolution of one cell and the average (black line) response (1000 cells). A–B Two binding sites. C–D Three binding sites. A and C: independent binding sites on the operator, B and D: with bridging.
(EPS)

Figure S4
**A higher affinity of TAT for the operator DNA and an increasing toxin translation rate cause more persister cell formation in the interacting binding sites model.** The number of toxin spikes with amplitude larger than 10, detected by analyzing the time evolution of 320000 cells during a time of 500 minutes each cell are plotted. In panel A, the binding site affinity of TAT to the DNA is changed. The red markers show the simulation results using the parameter set as shown in Table 1 of the article, while the green and blue markers correspond to a 10 fold decrease and increase in the binding affinity, respectively (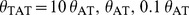). In panel B, the effect of changing the toxin translation rate is investigated. The red markers again show the simulation results using the parameter set as shown in Table 1 of the article, while the green and blue markers correspond to a 3 fold decrease and increase in the toxin translation rate, respectively. Depending on the parameters, two characteristic scaling laws are found. The first one is related to regular stochastic variation, and a second one with lower probability is related to rare events where a TAT complex stays bound on the DNA for a limited time determined by the DNA binding affinity of TAT.(EPS)

Figure S5
**Depending on the toxin and antitoxin translation rates, persister cells occur in the model with interacting binding sites on the operator.** Parameter scans for 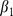 versus 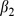 showing the percentage of cells (out of 200 simulated cells) that reach a free toxin level higher than 100 during a time of 500 minutes, both for independent and interacting binding sites. Time series exemplifying the behavior in each characteristic region are shown in panels (A) and (B). Panel A shows a cell with a controlled toxin level in the case of independent binding sites and the formation of a persister cell, a rare stochastic event which can occur in the case of interacting binding sites. Panel B shows that in the region [K], the toxin level in the cell keeps on growing and thus reaches a fatal concentration. The value of 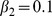 and 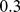 in (A) and (B), respectively, while 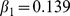 in both cases.(EPS)

Figure S6
**The effect of toxic feedback inclusion to the independent binding site model with two sites.** The effect of the feedback on the time evolution for 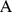 and 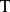 is shown in panel A with feedback (dashed line) and without (solid line). Panel B shows the corresponding p.d.f. for 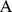 and 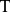, and corresponds to the plots shown in in Figure 3 (also simulated for 500 min and for 1000 cells). In panel C is the fitness landscape as in figure 7D, but obtained from 1 cell simulated over 100 days. The four panels have the standard parameters, with 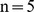, 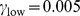 and the threshold 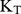 is changed as indicated in the legend with the calculated R.(EPS)

Figure S7
**Increasing antitoxin degradation rates cause increasing amounts of free toxin.** Parameter scans for 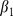 versus 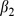 show the free toxin T level in the case of independent binding sites on the operator and this for different values of the degradation rate of antitoxin. The response of 200 cells has been averaged after simulating for 500 minutes. In the region [K], indicated in gray, the free toxin level grows continuously to large numbers, corresponding to a a non-culturable cell population.(EPS)

Figure S8
**Higher antitoxin degradation rates and lower growth rates are responsible for the increase in the amount of persisters during nutritional stress.** Panel A–E describe the situation for the normal parameter set (A) and three effects associated with nutritional stress: decreasing translation rates (B), increasing antitoxin degradation rates (C), decreasing growth rates (D) and their combination (E). The left panels show a probability distribution of the fitness landscape in each of these cases, the right panels present the correlation between spike duration and spike amplitude. The corresponding normalized average growth rate R and transfer rates from normal to persister state are shown. 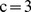, 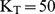, 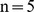 and 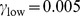.(EPS)

Text S1 **Supporting information to the article.**
(PDF)
