# Supplementary material for: A General Model for Toxin-Antitoxin Module Dynamics Can Explain Persister Cell Formation in E. coli
Source: PLoS Comput Biol. 2013 Aug 29;9(8):e1003190. doi: 10.1371/journal.pcbi.1003190 (PMC3757116; doi:10.1371/journal.pcbi.1003190)
Supplement: Text S1 — Supporting information to the article. (PDF) [file pcbi.1003190.s009.pdf]

Supporting Information

Figure S1

Sequestration in complexes, rather than gene regulation, controls the free toxin levels. We performed a series of simulations in which either operator binding or the sequestration of the toxin in complex TAT or in both non-toxic complexes (AT and TAT) were eliminated. Figure S1 shows the single cell response and an average response of 1000 cells under different circumstances. The green dotted line is the simulation result without any protein binding to the DNA promoter/operator site. The red line excludes sequestration of toxin into the complex TAT, while the light blue line excludes sequestration of toxin in any complex (both AT and TAT).

From this figure it can be seen that when DNA binding for A and AT is excluded, the simulation shows a controlled toxin level. This means that sequestration of the toxin into the complexes (AT and TAT) is responsible for the suppression of the free toxin level. The other simulations performed focussed on the formation of these complexes with the DNA binding included. In one of the cases the creation of the complexes AT and TAT was completely excluded and in the other just the TAT creation (and by extension conditional cooperativity) was eliminated. This showed that while the AT formation is necessary for toxin control the TAT formation helped to reduce the variability in the antitoxin level.

Figure S2

As the number of binding sites on the operator increases, the response becomes more localized in time. Figure S2 shows the effect of the number of independent binding sites on the operator on the time evolution of the antitoxin and toxin level and the binding on the DNA. The probability of the DNA operator region being unbound decreases with increasing number of binding sites. Furthermore, binding and unbinding events become less frequent in the model with larger numbers of binding sites on the operator. The plots with four binding sites show more localized bursts in time of antitoxin and toxin and these correspond to spikes in the mRNA level. Moreover, it can clearly be seen that increasing the number of binding sites on the operator leads to decreased protein levels for the free antitoxin, while the free toxin level stays low and relatively constant.

Figure S3

Regular oscillatory behavior is observed in the model with interacting binding sites. In Figure S3, the time evolution of the antitoxin is shown for a single cell, together with the average over 1000 cells using different models for the binding sites on the DNA. As mentioned before, an increase in the number of independent binding sites on the operator leads to protein spikes that are more localized in time and the average protein level decreases linearly with the number of binding sites (Figure S3A and C). In the case of interacting binding sites on the operator, that is in the presence of bridging between binding sites (Figure S3B and D), these results still hold. Moreover it is clear from Figure S3 that the addition of this dependency between binding sites on the DNA leads to a much more regular oscillatory behavior. Even when considering the average response taken over 1000 cells (black line), a clear fixed period is observable after which a spike can be anticipated. In contrast, in the case of independent binding sites on the operator, the average response is flat due to the irregularity of the spikes in the protein level.

Supplementary Material: Analytical calculation for the boundary between toxin dominated and antitoxin dominated bacterial populations

If the toxin translation rate exceeds twice the antitoxin translation rate, free toxin accumulates. In Figures 5 and 6 we observed that whenever the toxin translation rate exceeds twice the

antitoxin translation rate, the free toxin population grows to very large protein levels, corresponding to a non-culturable cell population. Here, we clarify analytically how this behavior can be understood. In the analysis below, we assume that $\theta_{\mathrm{TH}} = 0$, which is reasonable as the formed complexes AT and TAT are formed with high affinity and thus very stable. Moreover, we neglect binding to the DNA operator site and focus on the dynamics of the interacting proteins under free translation (DNA operator site unbound). Under these conditions, we can write down the following set of ODE equations describing the system:

$$\frac{d[M]}{dt} = \rho_F D - d_m[M], \tag{1}$$

$$\frac{d[A]}{dt} = \beta_1[M] - \alpha_{TH}[A][T] - d_a[A], \tag{2}$$

$$\frac{d[T]}{dt} = \beta_2[M] - \alpha_{TH}[A][T] - \alpha_{TH}[AT][T] - d_c[T], \tag{3}$$

$$\frac{d[AT]}{dt} = \alpha_{TH}[A][T] - \alpha_{TH}[AT][T] - d_c[AT], \tag{4}$$

$$\frac{d[TAT]}{dt} = \alpha_{TH}[AT][T] - d_c[TAT]. \tag{5}$$

Looking for steady state solutions for M, AT and TAT, one easily finds that

$$[M] = \frac{\rho_F D}{d_m}, \tag{6}$$

$$[AT] = \frac{\alpha_{TH}[A][T]}{\alpha_{TH}[T] + d_c} \approx [A], \tag{7}$$

$$[TAT] = \frac{\alpha_{TH}[AT][T]}{d_c}, \tag{8}$$

where we have assumed that $\alpha_{\mathrm{TH}}[\mathrm{T}] >> \mathrm{d_c}$. Using Eq. (7), we find a simplified system governing the dynamics of the toxin and antitoxin:

$$\frac{d[A]}{dt} = \beta_1[M] - \alpha_{TH}[A][T] - d_a[A], \tag{9}$$

$$\frac{d[T]}{dt} = \beta_2[M] - 2\alpha_{TH}[A][T] - d_c[T], \tag{10}$$

Simulations point towards the existence of two regions of operation, Region I where the antitoxin dominates the toxin and Region II where the reverse is true (the toxin level is much larger than the antitoxin level, also labeled by region [K] in Figures 5 and 6). The steady state levels $[\mathrm{A}] = \mathrm{A_S}$, $[\mathrm{T}] = \mathrm{T_S}$ in both regions can be approximated as follows:

Region I: $\mathrm{T_S} \approx 0$, $[\mathrm{A}] = \mathrm{A_S}$

$$A_S \approx \frac{\beta_1[M]}{d_a}, \tag{11}$$

$$T_S \approx \frac{\beta_2 d_a}{2\beta_1\alpha_{TH}}. \tag{12}$$

Region II: $[\mathrm{T}] = \mathrm{T_S}$, $\mathrm{A_S} \approx 0$

$$A_S \approx \frac{\beta_1 d_c}{\beta_2\alpha_{TH}}, \tag{13}$$

$$T_S \approx \frac{\beta_2 M}{d_c}. \tag{14}$$

In order to locate the crossover between both characteristic regions, we look for the conditions under which the steady state levels of toxin and antitoxin are equal ($\mathrm{A_S} = \mathrm{T_S}$). Using the fact that $\mathrm{d_a} << \alpha_{\mathrm{TH}}$ and $\mathrm{d_c} << \alpha_{\mathrm{TH}}$, one finds from Eqs. (9)-(10):

$$A_S \approx \frac{\beta_1[M]}{\alpha_{TH} T_S}, \tag{15}$$

$$T_S \approx \frac{\beta_2[M]}{2\alpha_{TH} A_S}, \tag{16}$$

such that $\mathrm{A_S} = \mathrm{T_S}$ when $\beta_2 \approx 2\beta_1$, which corresponds to our numerical findings. This can easily be understood, as the toxin needs to be produced twice as fast as the antitoxin before the free toxin level can grow because a single antitoxin is able to sequester two toxins.

Figure S4

A higher affinity of TAT for the operator DNA and an increasing toxin translation rate cause more persister cell formation in the interacting binding sites model. Figure S4 shows detailed simulations of toxin frequency and amplitude with reference to the unbinding rate of the complex TAT (θ_{TAT}) from the DNA (Panel A) and the toxin translation rate (Panel B). For both simulations the red markers show the response using the parameters listed in Table 1 in the main article. There are two observable characteristic scales. The first one is associated to regular stochastic fluctuations of the toxin amplitude under normal operation. The probability of finding toxin spikes of increasingly high amplitude decreases exponentially. The second scaling can be attributed to the different mechanism where a TAT complex remains bound to the DNA for a certain time, as mentioned above. Panel A of Figure S4 shows that as one decreases the binding affinity of TAT to the DNA operator site below a critical threshold, the second scaling corresponding to rare high amplitude toxin spikes disappears. Panel B demonstrates that when increasing (decreasing) the toxin translation rate β_2, toxin spikes become more (less) common and of higher (lower) amplitude.

Figure S5

Depending on the toxin and antitoxin translation rates, persister cells occur in the model with interacting binding sites on the operator. The figure shows a comparison between the model with three independent binding sites and the three interacting binding sites model. Each column presents a parameter scan of the two translation rates (β_1 and β_2) and a plot showing the toxin and antitoxin levels for the given points A and B. Point A represents the parameter values showing controlled toxin level in the cell and point B represents typical behavior within the region [K]. Since the toxin level at this latter point continuously grows this reflects an unviable situation for the cell - although in this simulation no toxic effect is included and so the response may not be the same *in vivo*.

As shown in Figure 5 (main article) there is a sharp transition between controlled and dying cells when the ratio of the translation rates for the antitoxin and toxin is 1:2. This boundary neatly divides the simulated cells into the two populations shown in the point A and point B examples. This boundary is more defined in the independent binding site case than for the interacting sites.

Another response found in the simulations for interacting binding sites on the operator was that of a toxin spike. This is a short lived pulse in the toxin level that quickly returns to the normal low operating level. This is a rare event which can be stochastically initiated if a TAT complex is bound to the DNA in the presence of very low levels of antitoxin and the complex AT. The probability of having toxin spikes, and therefore the potential of persisters occurring in the population, increases as one approaches the $\beta_2 = \beta_1$ line. The toxin spike becomes increasingly high with increasing values of β_2 (see Figure S4). In the region $\beta_1 < \beta_2 < 2\beta_1$ every cell will reach toxin levels higher than 100, but the response can either be a toxin

spike as shown in panel A or a continuously growing toxin level as shown in panel B. The percentage of the cells responding with continuously growing toxin levels increases (to 100%) as one approaches the $\beta_2 = 2\beta_1$ line.

Figure S6

The effect of toxic feedback inclusion to the independent binding site model with two sites. In the independent binding site model the toxic feedback effects have only marginal impact. No long-term persister dynamics are found using this model (Figure S6). The two panels (A and B) show that the addition of toxic feedback effects has marginal effect since in panel A the time traces including and excluding toxic effects are similar and the probability distribution functions with toxic feedback shown in Panel B are comparable to Figure 3 (two binding sites), excluding these feedback effects. The bottom panel in this figure shows the effect of the threshold level chosen in the Hill function and its impact on the overall normalized growth rate. When the threshold $\mathrm{K_T}$ is high, no decrease in the growth rate is observed as the free toxin levels are too low to cross the threshold. As this threshold is lowered, lower growth rates can be observed and R decreases. For this model there is no visible bimodality and the normalized growth rate does not reach its lower boundary $\gamma = \gamma_{\mathrm{low}}$. Therefore, no clear switch to a persister state is seen in this case.

Figure S7

Increasing antitoxin degradation rates cause increasing amounts of free toxin. Two parameters which change during nutritional stress are the antitoxin degradation rate and the rate of protein synthesis. Figure S7 shows that the boundary $\beta_2 = 2\beta_1$ for the viability of a cell population does not change when increasing the antitoxin degradation rate. However, in the antitoxin dominated region, this increase in the antitoxin degradation does cause an increase in the average free toxin level, which is typically associated with nutritional stress. A simultaneous decrease in the toxin and antitoxin translation level does not have a large effect on the free toxin level.

Figure S8

Higher antitoxin degradation rates and lower growth rates are responsible for the increase in the amount of persisters during nutritional stress. We investigate how the increase in antitoxin degradation and the decrease in translation rates and in growth rates associated with nutritional stress affect the formation of persister cells. We again use the model by Balaban *et al.* [1], describing a switch from normal growth to persistence:

$$\frac{dN}{dt} = -aN + bP + \mu_N N \tag{17}$$

$$\frac{dP}{dt} = aN - bP + \mu_P P \tag{18}$$

where N and P are the two states of the model: normal (N) and persister (P) and the switching rate from N to P and P to N are defined as a and b, respectively, while the growth rate of both states are given by μ_N and μ_P. These growth rates correspond to the peaks in the bimodal fitness distribution ($\mu_N = d_c$, $\mu_P = d_c\gamma_{low}$).

This model was used to analyze the fitness landscape of cell populations when changes in growth, translation and antitoxin degradation occur, by estimating the switching rates a and b from our simulations

(Figure S8). We looked at each of these effects separately (B, C and D) and then combined (E) in order to see how they influence the persister generation. Using the standard parameter set no clear, well-separated persister population is found in the scatterplot in Figure S8A. A decrease in the translation rate with a factor two does not cause the emergence of persisters (Figure S8B). However, when the antitoxin degradation is doubled or the growth rate halved, there are two distinct populations of cells (Figure S8C and S8D): in addition to the population dividing at a normal growth rate, there is a fraction dividing at a normalized growth rate γ_{low}. Both the modulation of the antitoxin degradation rate and the growth rate decrease the average fitness R, although the former has a larger effect than the latter. The switching rates for both these changes are similar and show that the transfer from normal to persister state occurs at a faster speed (a >b) than the return. In Figure S8E, we include all three rate modulations. This case is markedly different from the normal case (Figure S8A), as the switching rate from the normal to the persister population is now significantly higher and R has decreased even further due to a combined effect of the three different modulations.

References

[1] Balaban NQ, Merrin J, Chait R, Kowalik L, Leibler S (2004) Bacterial persistence as a phenotypic switch. Science 305: 1622-1625.
